# Linkage of Marine Bacterial Polyunsaturated Fatty Acid and Long-Chain Hydrocarbon Biosynthesis

**DOI:** 10.3389/fmicb.2019.00702

**Published:** 2019-04-03

**Authors:** Marco N. Allemann, Christine N. Shulse, Eric E. Allen

**Affiliations:** ^1^Marine Biology Research Division, Scripps Institution of Oceanography, University of California, San Diego, La Jolla, CA, United States; ^2^Center for Marine Biotechnology and Biomedicine, Scripps Institution of Oceanography, University of California, San Diego, La Jolla, CA, United States; ^3^Division of Biological Sciences, University of California, San Diego, La Jolla, CA, United States

**Keywords:** hydrocarbon, thioesterase, omega-3 polyunsaturated fatty acid, *Shewanella*, *Photobacterium*

## Abstract

Various marine gamma-proteobacteria produce omega-3 polyunsaturated fatty acids, such as eicosapentaenoic acid (20:5, EPA) and docosahexaenoic acid (22:6, DHA), which are incorporated into membrane phospholipids. Five genes, designated *pfaABCDE*, encode the polyketide/fatty acid synthase necessary for production of these long-chain fatty acids. In addition to *de novo* biosynthesis of EPA and DHA, the “Pfa synthase” is also involved with production of a long-chain polyunsaturated hydrocarbon product (31:9, PUHC) in conjunction with the *oleABCD* hydrocarbon biosynthesis pathway. In this work, we demonstrate that OleA mediates the linkage between these two pathways *in vivo*. Co-expression of *pfaA-E* along with *oleA* from *Shewanella pealeana* in *Escherichia coli* yielded the expected product, a 31:8 ketone along with a dramatic ∼10-fold reduction in EPA content. The decrease in EPA content was independent of 31:8 ketone production as co-expression of an OleA active site mutant also led to identical decreases in EPA content. We also demonstrate that a gene linked with either *pfa* and/or *ole* operons in diverse bacterial lineages, herein designated *pfaT*, plays a role in maintaining optimal production of Pfa synthase derived products in *Photobacterium* and *Shewanella* species.

## Introduction

**FIGURE 1:**
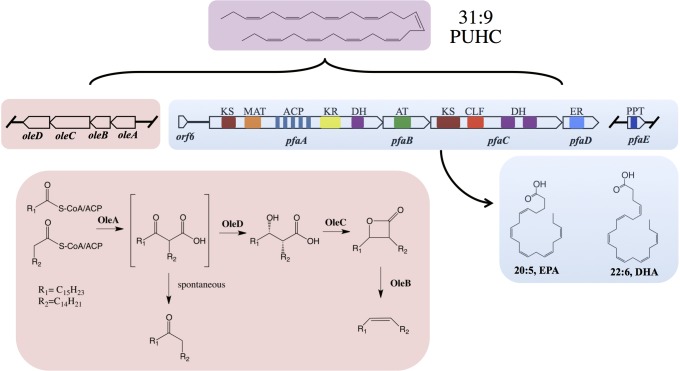
Biochemical and genetic aspects of PUFA and PUHC biosynthetic pathways. Domain designations within the Pfa synthase are; phosphopantetheinyl transferase (PPT), β-ketoacyl synthase (KS), malonyl-CoA:ACP transacylase (MAT), acyl-carrier protein (ACP), ketoacyl reductase (KR), dehydratase/isomerase (DH), acyltransferase (AT), chain-length factor (CLF), and enoyl reductase (ER). Both *pfaE* and/or *oleABCD* can be clustered with the *pfa* operon or found elsewhere in the genome depending on the host organism.

The pathway for biosynthesis of omega-3 polyunsaturated fatty acids (PUFAs) in certain marine bacteria occurs via a polyketide synthase type mechanism encoded by five genes *pfaABCDE* (Metz et al., 2001; Allen and Bartlett, 2002; Shulse and Allen, 2011; Yoshida et al., 2016; Allemann and Allen, 2018). The “Pfa synthase” has been identified in several bacterial lineages and shown to synthesize a variety of PUFA products, most notably the long-chain omega-3 PUFAs, such as eicosapentaenoic acid (20:5*n*-3, EPA) and docosahexaenoic (22:6*n*-3) acids (Shulse and Allen, 2011; Yoshida et al., 2016; Allemann and Allen, 2018). The Pfa synthase multienzyme complex contains all of the required enzymatic domains for lipid biosynthesis and these activities reside on either multi-domain or stand-alone proteins (Figure 1). Recombinant production of EPA or DHA in *Escherichia coli* has been demonstrated by heterologous expression of *pfaA-E* from various marine bacterial strains (Metz et al., 2001; Okuyama et al., 2007; Amiri-Jami and Griffiths, 2010). Akin to canonical fatty acid biosynthesis, PUFAs are synthesized from 2C malonyl extender units (Metz et al., 2001) and the final product is incorporated into membrane phospholipids of the producing strain (Yazawa, 1996; Yoshida et al., 2016).

The biosynthesis of long-chain fatty acid-derived olefin hydrocarbons has been previously shown to be the result of the *oleABCD* pathway in various marine and non-marine bacterial lineages (Beller et al., 2010; Sukovich et al., 2010a,b). In this pathway, hydrocarbons are generated by the condensation of two fatty acyl chains via a “head-to-head” Claisen condensation mechanism, resulting in an intermediate β-keto acid product (Figure 1; Frias et al., 2011; Goblirsch et al., 2016). After condensation, the β-keto acid produced by OleA is processed by OleD, a NADPH-dependent reductase, to form a β-keto alcohol (Bonnett et al., 2011). OleC, a β-lactone synthetase, then generates a β-lactone moiety (Christenson et al., 2017b) which is subsequently de-carboxylated by OleB, a β-lactone decarboxylase, yielding the final olefin product (Christenson et al., 2017c). Recent work has demonstrated that OleBCD together form a large multimeric enzyme complex that processes the β-keto intermediate formed by OleA activity (Christenson et al., 2017a). Weak interactions between the OleBCD complex and OleA *in vitro* suggests that OleA condenses the precursor acyl groups and possibly transfers the β-keto acid product directly to the OleBCD complex for further processing (Christenson et al., 2017a). In the absence of downstream processing by OleBCD, the OleA catalyzed β-keto acid intermediate undergoes spontaneous decarboxylation to form a ketone product (Sukovich et al., 2010b; Frias et al., 2011; Figure 1).

The precursor fatty acids for olefin biosynthesis are derived from the cellular fatty acid biosynthesis pathway(s) in the producing host (Sukovich et al., 2010a). In strains such as *Xanthomonas campestris* and *Stenotrophomonas maltophilia*, the C27–C31 olefin products are derived from the core Type II fatty acid synthase (FAS), which produces saturated, monounsaturated, or branched chain fatty acids (Beller et al., 2010; Sukovich et al., 2010a). However, marine bacteria that synthesize EPA and/or DHA via the Pfa synthase mechanism produce a unique polyunsaturated hydrocarbon (Nichols et al., 1995), hentriacontanonaene (31:9, PUHC), via the OleABCD pathway (Sugihara et al., 2010; Sukovich et al., 2010a,b). Previous genetic experiments in *Shewanella oneidensis* MR-1 verified that PUHC biosynthesis is dependent on the Pfa synthase, as mutations in the *pfa* operon led to loss of PUHC production (Sukovich et al., 2010b). Interestingly, despite the presence of the Type II FAS in PUFA-producing bacteria, OleA exclusively condenses a predicted 16:4*n*-3 acyl chain derived from the Pfa synthase in these species.

Given the biosynthetic linkage of the *ole* and *pfa* pathways, it is not surprising that in some strains such as *Photobacterium profundum* SS9, the two operons are located adjacent to one another on the chromosome (Allen and Bartlett, 2002; Vezzi et al., 2005). Intriguingly, a previously characterized gene encoding an acyl-CoA thioesterase, previously designated *orf6*, sits between the two operons in *P. profundum* SS9. Previous structural and biochemical characterization of Orf6 revealed a “hot dog” fold topology with thioesterase activity on various long-chain acyl-CoA substrates *in vitro* (Rodríguez-Guilbe et al., 2013). Based on its activity and its conservation among EPA/DHA producing bacteria, it was speculated that this thioesterase may be involved with product release from the Pfa synthase (Rodríguez-Guilbe et al., 2013). However, modest rate enhancement of thioesterase activity *in vitro* raised doubts as to the role of Orf6 in product release (Rodríguez-Guilbe et al., 2013).

In this work we establish the linkage between PUFA and PUHC biosynthesis and show that OleA is responsible for mediating the linkage between the two pathways. Our results indicate that OleA can interact with the Pfa synthase directly, most likely with the acyl carrier protein (ACP) domains that shuttle acyl intermediates, including the 16:4*n*-3 PUHC precursor molecules, among catalytic domains during *de novo* PUFA biosynthesis. We also investigated the *in vivo* role of the *orf6* gene in *P. profundum* SS9 and *S. oneidensis* MR-1, demonstrating that it is required for optimal biosynthesis of EPA or PUHC in each strain, respectively. Given our results, we have re-designated *orf6* as *pfaT* (*pfa*-associated thioesterase). Together, these results provide new insight into the genetic and enzymatic determinants involved in the bacterial synthesis of long-chain fatty acid and hydrocarbon products of biotechnological interest.

## Materials and Methods

### Bacterial Strains and Growth Conditions

A list of strains used in this study is shown in Table 1. *E. coli* and *S. oneidensis* MR-1 strains were cultured in Luria Bertani media at 37 and 30°C, respectively, unless noted otherwise. *P. profundum* SS9R, a rifampin-resistant derivative of wild-type SS9, and *Shewanella pealeana* strains were cultured in 75% strength 2216 marine broth media (BD Difco, 28 g/L) at 15°C unless noted otherwise. For high hydrostatic pressure growth studies, SS9R strains were grown in heat-sealed bulbs as described previously (Chi and Bartlett, 1993). For analysis of heterologous production of EPA in *E. coli*, relevant strains were grown at 15°C for 48 h. For solid medias, agar was included at 15 g/L. The antibiotics kanamycin (50 μg/ml for *E. coli* and *S. oneidensis* MR-1; 200 μg/ml for *P. profundum*), chloramphenicol (15 μg/ml), carbenicillin (100 μg/ml), and rifampicin (100 μg/ml) were used as required.

**Table 1:** Strains and plasmids used in this study.

Strain	Genotype or relevant characteristics	Source
***Shewanella* strains**		
*S. pealeana* ATCC 700345	Wild type, EPA^+^, PUHC^+^	American Type Culture Collection
*S. oneidensis* MR-1	Wild type, EPA^-^, PUHC^+^	Heidelberg et al., 2002
*S. oneidensis* Δ*ole*	Δ*oleABCD*, EPA^-^, PUHC^-^	Sukovich et al., 2010a
*S. oneidensis* MAS1	Δ*orf6*	This study
***Photobacterium* strains**		
SS9R	Rifampicin resistant, EPA^+^, PUHC^-^	Chi and Bartlett, 1993
MAP1	SS9R, Δ*pfaT*	This study
***E. coli* strains**		
DH5α*pir*	Cloning strain, maintaining R6K plasmids	Saltikov and Newman, 2003
WM3064	Conjugal donor strain used for MR1	Saltikov and Newman, 2003
S17-1λ*pir*	Conjugal donor strain used for SS9	Simon et al., 1983
BW25113	Keio collection parental strain	Coli Genetic Stock Center
JW1794	Keio collection Δ*fadD::kan*	Coli Genetic Stock Center
MAE21	JW1794, 1F12R	This study

**Plasmids**	**Relevant characteristics**	**Source**

pBAD24	Arabinose inducible expression vector, Amp^R^	Guzman et al., 1995
pRE118	R6K origin allelic exchange plasmid, Kan^R^, SacB	Edwards et al., 1998
pRK2073	Contains *tra* genes for conjugal transfer	Better and Helinski, 1983
pKT231	Complementation plasmid for SS9, Kan^R^ Sm^R^	Bagdasarian et al., 1981
pCC2FOS	Copy control fosmid, Cm^R^	Epicentre
1F12R	pCC2FOS containing *pfaA-E* from *S. pealeana*	This study
pOleA	pBBR1MCS-2 with containing *oleA* from *S. oneidensis*	Sukovich et al., 2010a
pMA10	pRE118 containing Δ*pfaT* allele for *S. oneidensis* MR-1, cloned as *Nde*I-*Sac*I fragment	This study
pMA12	pRE118 containing Δ*pfaT* allele for *P. profundum* SS9, cloned as *Kpn*I-*Sac*I fragment	This study
pMA20	pKT231 containing *pfaT* region from *P. profundum* SS9, cloned as *Bam*HI-*Eco*RI fragment	This study
pMA47	pBAD24 containing *pfaT* from *P. profundum* SS9, cloned as *Eco*RI-*Xba*I fragment	This study
pMA48	pBAD24 containing *pfaT* from *S. pealeana*, cloned as *Eco*RI-*Pst*I fragment	This study
pMA63	pBAD24 containing *oleA* from *S. pealeana*, cloned as *Nhe*I-*Pst*I fragment	This study
pMA70	pBAD24 containing C123A OleA, mutant derived from pMA63	This study


*Amp, ampicillin; Cm, chloramphenicol; Kan, kanamycin; Sm, Streptomycin*.

### Gene Disruption Mutagenesis

To generate in-frame deletions of genes, an allelic exchange approach was used similar to previous work (Eloe et al., 2008). Briefly, upstream and downstream regions of the gene of interest were amplified with the appropriate primer combinations (5′O, 5′I) and (3′O, 3′I), respectively (Supplementary Table 1). Purified PCR products were assembled using overlap PCR and subsequently amplified with 5′O and 3′O primers. Assembled fragments were then cloned into the suicide vector pRE118 (Edwards et al., 1998) using standard methods (Sambrook et al., 1989). Colony PCR and subsequent DNA sequencing were used to verify constructs.

For conjugation into *P. profundum*, biparental matings were performed using the *E. coli* donor strain S17-1λ*pir* containing the desired plasmid to be mobilized into SS9R. Selection for exconjugants was performed on 2216 agar containing rifampicin and kanamycin as described previously (Lauro et al., 2005; Allemann and Allen, 2018). After colony purification of exconjugants, colonies were grown without selection and dilutions plated onto 2216 agar supplemented with 5% sucrose to select for a second recombination event. Colony PCR was used to screen sucrose resistant clones for the targeted deletion.

For conjugation into *S. oneidensis* MR-1, biparental matings were performed using an *E. coli* diaminopimelic acid (DAP) auxotroph, WM3064, on LB agar supplemented with DAP, as described previously (Yang et al., 2015). Exconjugants were selected on LB plates containing kanamycin without DAP. Colony purified exconjugants were grown in LB without NaCl for several generations and subsequently plated onto LB supplemented with 5% sucrose for counter-selection. Sucrose resistant colonies were screened for kanamycin sensitivity and colony PCR was used to screen kanamycin sensitive clones for the targeted deletion.

### Cloning/Expression Procedures

The *pfa* operon from *S. pealeana* was cloned into the pre-linearized pCC2FOS vector following manufacturer guidelines (Epicentre, Madison, WI, United States). Briefly, a fosmid library was constructed from *Swa*I digested genomic DNA from *S. pealeana*. Colony PCR using primers listed in Supplementary Table 1 specific to *pfaD* and *pfaE* was used to screen clones for presence of the *pfa* operon. A single clone, which contained the entire cluster was identified and designated 1F12R. Other plasmids were constructed by PCR amplification of indicated genes using the appropriate primer pair containing restriction sites (i.e., pBAD24 SS9 orf 6 F/R) listed in Supplementary Table 1. PCR products were cloned into various plasmids at restriction sites given in Table 1 using standard procedures (Sambrook et al., 1989). Site-directed mutagenesis of OleA was accomplished using PCR mutagenesis primers listed in Supplementary Table 1. Briefly, the plasmid pMA63 was used as a template for PCR using mutagenesis primer pair “OleA C123A mut F/R.” A restriction digest using *Dpn*I removed the original plasmid template and the resulting DNA was transformed into competent cells. DNA sequencing was used to verify the introduced mutation. For expression of genes cloned onto pBAD24 (Guzman et al., 1995) L-arabinose was added. Expression of OleA and Orf6 (PfaT) homologs was confirmed by SDS-PAGE of whole cell lysates followed by Coomassie staining (Sambrook et al., 1989).

### Fatty Acid/Neutral Lipid Extraction and Analysis

Late log phase cultures were harvested by centrifugation and cell pellets rinsed once with 50% Sigma Sea Salts solution (16 g/L) and stored at -80°C. Cell pellets were lyophilized prior to fatty acid or hydrocarbon analysis.

For fatty acid analysis, lipids were converted to fatty acid methyl esters (FAME) by adding 5% H_2_SO_4_ in methanol directly to lyophilized biomass and refluxing at 90°C for 90 min. After cooling, hexanes were added and non-esterified fatty acids were saponified by addition of 10% NaCl. The hexane extraction was repeated twice and pooled fractions were evaporated completely under a gentle N_2_ stream and re-dissolved in 1 ml of hexane. Samples were stored at -80°C until analysis.

Hydrocarbon/ketones were extracted from freeze-dried biomass by addition of a mixture of dichloromethane/methanol (2:1, vol/vol) and stirred overnight at room temperature. The crude extract was filtered using a Pasteur pipet packed with glass wool and celite and subsequently dried under a gentle stream of N_2_ gas. A mixture of hexanes and methanol (4:1, vol:vol) were added to the residue and allowed to form a two-phase mixture. The hexane phase was removed and two additional hexane volumes were added to the methanol phase and subsequently extracted. The hexane fractions were pooled, dried under a stream of N_2_ and dissolved in 1 ml of hexane. For quantitative analysis hentriacontane (31:0) (Sigma) was added as an internal standard.

Gas chromatography mass spectrometry (GC-MS) analyses were performed on an Agilent Technologies model 7890A GC connected to a 5975C VL MSL quadrupole MS(EI). Samples were separated on a 30 m HP5ms Ultra Inert Agilent GC-MS column using helium as carrier gas. Fatty acid samples were injected in splitless mode and held at 110°C for 3 min followed by a gradient of 15°C/min, and held at a final temperature of 280°C for an additional 3 min. For analysis of neutral lipid extracts, samples were injected in splitless mode and held at 100°C for 3 min followed by a gradient of 15°C/min and held at a final temperature of 300°C for an additional 10 min. Both injector and detector for the mass spectrometer were maintained at 250°C. Additional MS operating conditions were as follows: mass range 50–500 atomic mass units, 3 min solvent delay. Peak areas were quantified and mass spectra processed using ChemStation software (Agilent Technologies). FAMEs were identified by comparing MS fragmentation patterns to spectra from authentic standards or from spectra on the NIST 2008 Spectral Library.

### Phylogenetic Analysis

Homolog amino acid sequences were obtained from a gene neighborhood search on the Joint Genome Institute Integrated Microbial Genome web portal (accessed on October 16, 2018). Sequences were uploaded to the Phylogeny.fr web portal and a maximum likelihood phylogenetic tree was constructed as described previously (Dereeper et al., 2008). For tree construction bootstrap values were generated from 100 re-samplings of the data.

## Results

**FIGURE 2:**
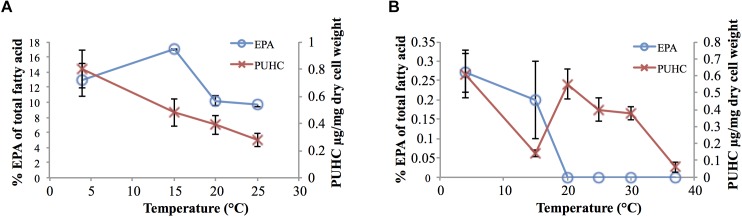
EPA and PUHC content as a function of temperature in **(A)** *S. pealeana* and **(B)** *S. oneidensis* MR-1. Error bars represent standard deviations based on three biological replicates.

Analysis of EPA and PUHC content in *S. pealeana* and *S. oneidensis* MR-1 as a function of growth temperature displayed the trend of both compounds increasing in abundance as temperature decreased (Figure 2). This is consistent with previous findings regarding EPA (Allen et al., 1999; Okuyama et al., 2007; Yoshida et al., 2016) and PUHC (Sukovich et al., 2010b) content as a function of growth temperature. Given the relationship between the *ole* and *pfa* pathways, increased flux through the Pfa synthase is predicted to result in a concurrent increase in PUHC production. Conversely, reducing flux into the *ole* pathway is predicted to lead to an increase in the *pfa* pathway. Given the relationship between the two pathways, it was hypothesized that previously observed differences in EPA content amongst members of the *Shewanella* genus (Kato and Nogi, 2001), may be due to the diversion of acyl substrates from the EPA pathway into the PUHC pathway. Deletion of the entire *ole* operon resulted in a modest ∼twofold increase in EPA content relative to wild-type *S. oneidensis* MR-1 at 15°C (Supplementary Table 2).

**FIGURE 3:**
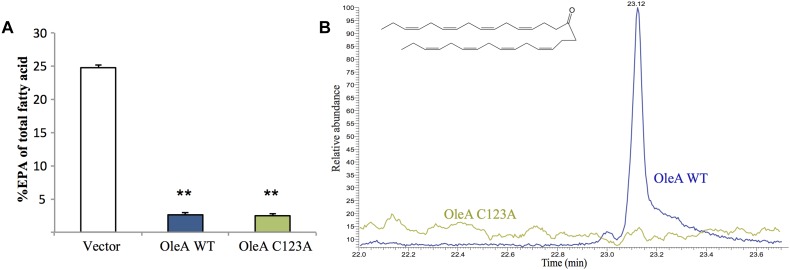
Expression of OleA in *E. coli* MAE21 strain harboring fosmid 1F12R with the *pfa* operon from *S. pealeana* leads to production of a 31:8 ketone and reduced EPA. **(A)** EPA content of MAE21 strains decreases upon induction of OleA^WT^ (pMA63) and OleA^C123A^ (pMA70) with 0.05% L-Arabinose. Bars represent averages of at least three biological replicates with error bars signifying one standard deviation (^∗∗^*P* < 0.005). **(B)** Total ion chromatograms (TIC) of neutral lipid extracts from MAE21 OleA^WT^ (pMA63 – blue) and MAE21 OleA^C123A^ (pMA70 – green). 31:8 ketone peak seen at 23.1 min.

To gain a better understanding of how the *ole* and *pfa* pathways interact, we performed combinatorial co-expression experiments in *E. coli*. The *pfa* operon was cloned from *S. pealeana* ATCC700345 onto pCC2FOS to yield the construct 1F12R. The *pfa* operon from *S. pealeana* was chosen due to its high EPA and PUHC production phenotypes in the native strain. Transformation of 1F12R into the *E. coli* strain JW1794 yielded strain MAE21 that produced EPA at ∼21% of total fatty acid when cultured at 15°C (Supplementary Table 3). The fatty acid profile of a control strain containing pCC2FOS vector only did not produce EPA. Given that OleA catalyzes the first committed step of hydrocarbon biosynthesis in the OleABCD pathway, we speculated that co-expression of OleA from *S. pealeana* might impact EPA production in MAE21. The OleA homolog in *S. pealeana* was successfully cloned under an arabinose inducible promoter (pMA63) and shown to produce a protein of the expected size (38 kDa) in MAE21 upon induction with L-arabinose (Supplementary Figure 1). Fatty acid analysis of the MAE21 strain co-expressing OleA (pMA63) indicated a dramatic ∼10-fold decrease in EPA content relative to the vector only control strain (Figure 3A). Attempts to titrate this effect with varying amounts of L-arabinose were unsuccessful (data not shown) and are most likely due to our inability to effectively titrate expression of the pBAD promoter under the growth conditions employed. Site-directed mutagenesis of the universally conserved OleA catalytic cysteine residue to alanine (C123A) was employed to determine if catalytically active OleA is required for this reduced EPA phenotype. Under identical conditions, co-expression of mutant OleA^C123A^ (pMA70) in MAE21 yielded nearly identical results as seen for OleA^WT^ (pMA63) (Figure 3A).

Previous work in *S. oneidensis* MR-1 had shown that expression of *oleA* alone without *oleBCD* led to production of a polyunsaturated ketone (31:8), which is the result of spontaneous decarboxylation of the OleA β-keto acid product (Figure 1; Sukovich et al., 2010a,b; Frias et al., 2011). Neutral lipid extracts of MAE21 containing OleA^WT^ (pMA63) and mutant OleA^C123A^ (pMA70) were analyzed by GC-MS. A peak at 23.1 min corresponding to the 31:8 ketone was found in the wild-type OleA (pMA63) containing strain but not in the mutant OleA^C123A^ (pMA70) containing strain (Figure 3B). Mass spectra associated with the peak at 23.1 min also matched the spectra of the 31:8 ketone produced by *S. oneidensis* Δ*ole* strain containing pOleA (Supplementary Figures 2A,B). MAE21 is a derivative of JW1794, which contains a Δ*fadD::kan* mutation, rendering the strain unable to produce acyl-CoA from free fatty acids (Cronan, 1997; Black and DiRusso, 2003). The appearance of the 31:8 ketone in the MAE21 strain background indicates that OleA can condense the appropriate acyl products in the absence of acyl-CoA synthetase activity.

**FIGURE 4:**
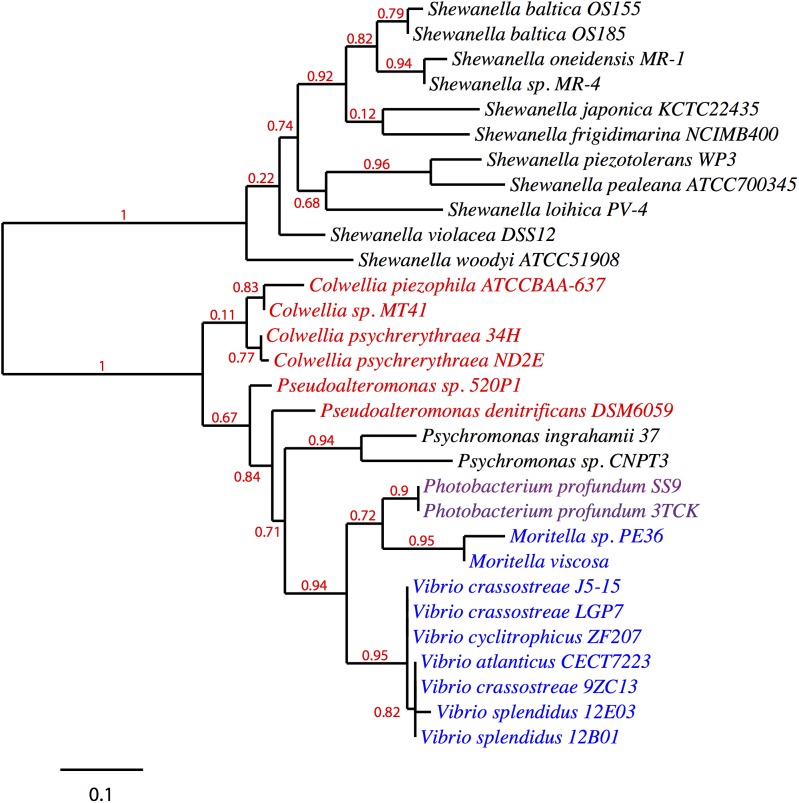
Phylogenetic distribution of PfaT homologs. A maximum likelihood unrooted tree of PfaT homologs found in bacteria with *pfa* and/or *ole* pathways. PfaT homologs clustered with the *pfa* operon are indicated in blue, homologs clustered with *ole* operon in red text, and clustering between the operons in purple. Numbers at branch points indicate bootstrap values based on 100 replicates.

Given its clustering with the *pfa* and/or *ole* operons in various EPA/DHA producing strains, *pfaT* (previously *orf6*) and its orthologs could be involved with one or both biosynthetic pathways. A search for homologs of *pfaT* was conducted and a protein sequence phylogenetic tree is shown in Figure 4. All of the included species are either known PUFA producers and/or contain both *pfa* and *ole* operons. With the exception of the *Shewanella* and *Psychromonas* species, all *pfaT* homologs were found to be genetically linked with either *pfa* or *ole* operons. All homologs shown in the tree contain the active site aspartate residue previously described to be essential for thioesterase activity (Rodríguez-Guilbe et al., 2013).

**FIGURE 5:**
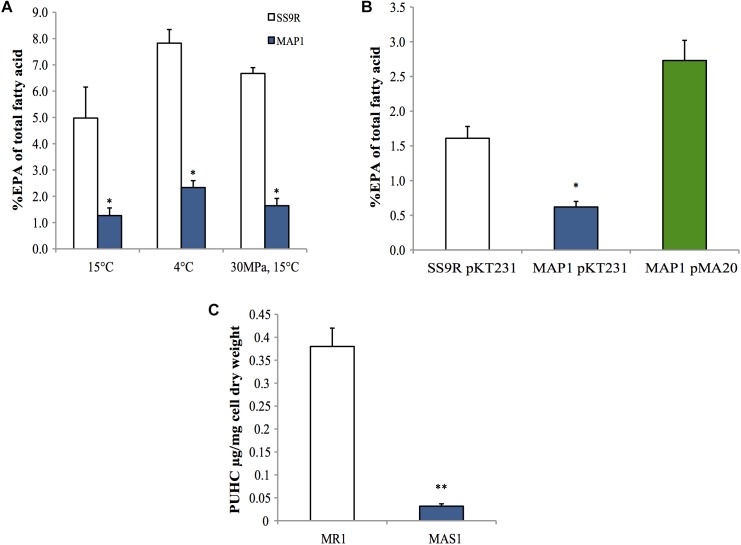
Phenotypes of *pfaT* deletion mutants in *P. profundum* SS9 and *S. oneidensis* MR-1. **(A)** An approximate fourfold decrease in EPA composition in *P. profundum* strain MAP1 harboring an in-frame deletion of *pfaT* relative to SS9R under various culturing conditions. **(B)** Complementation analysis. Comparison of the amount of EPA as a percentage of total fatty acids among parent strain SS9R harboring empty complementation plasmid pKT231, *P. profundum* strain MAP1 harboring empty complementation plasmid pKT231, and *P. profundum* strain MAP1 harboring plasmid pMA20 containing *pfaT* under control of its native promoter. Cells grown at 15°C as in **A**. Bars represent averages of at least three experimental replicates and error bars represent one standard deviation. **(C)** PUHC content of *S. oneidensis* strain MAS1 (harboring an in-frame deletion of the *pfaT* homolog) and parent strain *S. oneidensis* MR-1 at 30°C. Bars represent averages of three biological replicates and error bars represent one standard deviation (^∗^*P* < 0.05; ^∗∗^*P* < 0.005).

A marker-less in-frame deletion of *pfaT* was generated in *P. profundum* SS9 and the resulting strain, MAP1, was analyzed for EPA content under a variety of culture conditions. As shown in Figure 5A, MAP1 produced ∼fourfold less EPA compared to its parental strain SS9R at 15°C. This reduction in EPA content was also seen during growth at low temperature (4°C) and at high hydrostatic pressure (30 MPa) (Figure 5A), culture conditions that elicit increased EPA content in wild-type SS9 (Allen et al., 1999). The decreased EPA phenotype was also complemented *in trans* by a construct (pMA20) containing *pfaT* under control of its native promoter (Figure 5B). Full fatty acid profiles of SS9R and MAP1 along with the corresponding genetically complemented strains and controls are given in Supplementary Tables 4, 5, respectively. The ability to complement the *pfaT* mutation in strain MAP1 also confirms that the observed decrease in EPA is due to the loss of *pfaT* and not a polar effect on transcription of the *pfa* operon. Co-expression of *orf6* from *P. profundum* SS9R (pMA47) or the homolog from *S. pealeana* (pMA48) in the recombinant EPA-producing *E. coli* strain MAE21 did not lead to alterations in EPA content or changes in overall fatty acid profile (Supplementary Table 3). Furthermore, expression of both homologs in strain MAE21 or JW1794 did not lead to accumulation of free fatty acids (data not shown).

Surprisingly, neither MAP1 nor SS9R produced PUHC at a detectable level despite the presence of the *oleABCD* operon (data not shown). Given our previous characterization of *S. oneidensis* MR-1 and its genetic tractability, we generated an in-frame deletion of the *pfaT* homolog (locus tag SO1256) in *S. oneidensis* MR-1 (MAS1). Neutral lipid extracts of this strain indicated a drastic ∼10-fold reduction in PUHC content relative to the parental strain under identical conditions (Figure 5C). Fatty acid profiles of *S. oneidensis* MR-1 and MAS1 grown at 15°C are shown in Supplementary Table 2 and a similar fourfold decrease in EPA was observed.

## Discussion

In this work we have demonstrated that bacterial PUFA and PUHC biosynthesis are linked and this linkage is mediated by OleA. Our initial work with *S. pealeana*, which produces both PUFA and PUHC products, indicated that culture conditions which lead to increases in PUFA lead to commensurate increases in PUHC. While the physiological role of PUHC remains obscure, this result suggests that PUHC may play an additional role in adaptation to cold and/or high-pressure environments, conditions that impact membrane physical structure, e.g., fluidity. The modest EPA content in the Δ*ole* strain (<1% of total fatty acid) indicates that other factors in the biosynthesis and incorporation of EPA into membrane phospholipids are responsible for the previously observed differences in EPA production amongst members of the *Shewanella* genus (Kato and Nogi, 2001).

Our results also indicate that the *in vivo* OleA substrate is an acyl-ACP and not an acyl-CoA as described previously (Frias et al., 2011; Goblirsch et al., 2016). Our heterologous expression system in *E. coli* demonstrated that co-expressing *oleA* along with *pfaABCDE* was sufficient for producing the expected 31:8 ketone previously observed in *S. oneidensis* MR-1 mutants (Sukovich et al., 2010a). The nearly identical reduction in EPA content associated with co-expression of wild-type OleA or the OleA^C123A^ mutant suggests that OleA interacts directly with the ACP domains of the Pfa synthase and that this reduction is not a result of acyl groups being removed from the synthase. Rather, the reduction in EPA production may instead reflect steric competition for the ACP domains between OleA and the various catalytic domains on the Pfa synthase. From a biosynthetic standpoint, such direct ACP interactions would be the most efficient method for obtaining acyl groups for OleA condensation. Other routes of diverting substrates such as thioester cleavage would require acyl-CoA synthetase activity and would consume ATP to regenerate the acyl-CoA needed for condensation by OleA. Previous work indicated that OleA and its homologs specifically act on acyl chains derived from either the Type II FAS or the Pfa synthase (Sukovich et al., 2010a; Frias et al., 2011). Protein–protein interactions between OleA and its cognate ACP may be the mechanism for this substrate specificity.

In this work the *in vivo* role of the previously characterized *orf6* thioesterase, now designated *pfaT*, was investigated. While *pfaT* was not completely essential to EPA biosynthesis in *P. profundum* SS9, it was required for wild-type production levels. Similarly, the *pfaT* homolog in *S. oneidensis* MR-1 was not essential to PUHC biosynthesis; rather it was required for optimal biosynthesis of PUHC. Reduction in both end products of the Pfa synthase indicates that PfaT is not involved in a process specific to either pathway alone. Rather, these results suggest that PfaT may function as a Type II thioesterase with activity upon the Pfa synthase. Type II thioesterases serve accessory roles in removing aberrant intermediates or starter units from polyketide synthases and their genetic disruption typically leads to a reduction in the final polyketide product (Kotowska and Pawlik, 2014). Intriguingly, co-expression of various *pfaT* homologs in the EPA-producing *E. coli* strain MAE21 did not lead to modulation in EPA production. Previous studies in *E. coli* have described accumulation of free fatty acids in the culture media or intracellularly in response to expression of acyl-CoA/ACP thioesterases, particularly in strains in which β-oxidation is non-functional (Cho and Cronan, 1995; Lennen et al., 2011; Zhang et al., 2011). In all instances, expression of *pfaT* homologs in MAE21 or JW1794, did not lead to any free fatty acid accumulation in the media or the appearance of novel fatty acids. This discrepancy between results from the heterologous host *E. coli*, wherein omission or co-expression of PfaT led to no changes in EPA production, and native strains in which genetic disruption of PfaT led to decreases in both EPA and PUHC suggests that PfaT performs a function specific to biosynthesis of PUFA and/or PUHC in the native producing strains only. The relatively low *in vitro* thioesterase activity of PfaT (Orf6) reported previously (Rodríguez-Guilbe et al., 2013), along with phenotypic data presented herein is suggestive of PfaT acting as a type II thioesterase.

**FIGURE 6:**
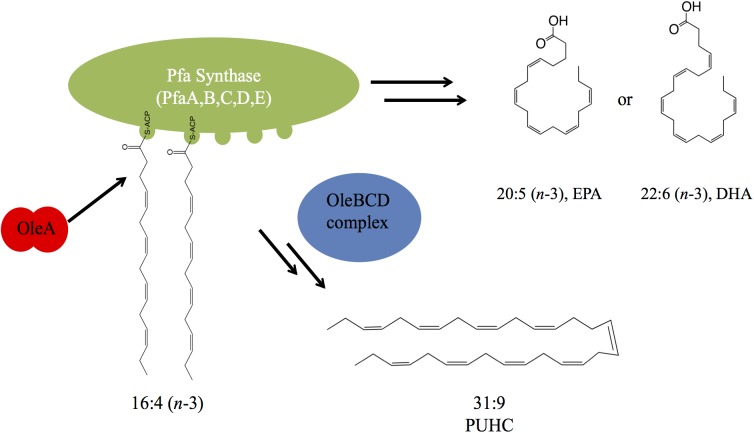
A model depicting the interaction between the Pfa synthase and Ole biosynthetic pathways. OleA interacts with the ACP domain(s) carrying a 16:4 acyl group. Condensation of two fatty acids is followed by further biosynthetic processing by the OleBCD complex to yield the 31:9 PUHC.

From the data presented, a model depicting the interaction between PUFA and PUHC biosynthesis is depicted in Figure 6. During EPA biosynthesis, a 16:4*n*-3 acyl group, a predicted intermediate of the biosynthetic pathway, is processed by OleA via direct interaction with the ACP domains on PfaA. After condensation, the β-keto acid product is further processed by the activities of the OleBCD complex to form the final 31:9 PUHC product (Figure 1). The absence of a significant increase in EPA production in the *S. oneidensis* Δ*oleABCD* strain relative to other members of the *Shewanella* genus indicates that diversion of intermediates to PUHC biosynthesis is not strictly responsible for the differences in EPA phenotypes observed previously (Kato and Nogi, 2001). The relative activities of the Pfa synthase between strains might be a possible mechanism for the observed differences in EPA production potential among *Shewanella* species.

OleA is a member of the thiolase protein family which have been shown to utilize either acyl-CoA and/or acyl-ACP substrates (Cronan and Rock, 2008; Goblirsch et al., 2012). Patent literature describing OleA from *S. maltophilia* demonstrated that OleA utilizes both acyl-CoA and acyl-ACP substrates *in vitro* (Friedman and Da Costa, 2008). Detailed examination of previously published crystal structures of OleA (Goblirsch et al., 2012, 2016) from *X. campestris* showed that the pantetheine channel entrance contains a cluster of positively charged residues, which form a “positive patch.” This positive patch feature is found in many fatty acid biosynthesis enzymes, which are known to interact with ACP (Zhang et al., 2001; Finzel et al., 2015). While there is no structural data corresponding to OleA homologs from any PUHC producers it is expected that a similar positive patch feature would be present.

Polyunsaturated fatty acids and PUHC biosynthesis are intrinsically linked and the results of this work have identified that OleA mediates this linkage *in vivo*. Notably, the results presented here suggest that OleA is capable of interacting with the Pfa synthase *in vivo* and that the acyl substrates are derived directly from the ACP domains of the synthase. This work has also more clearly defined the role of PfaT, not as a thioesterase for final product release, but rather as an accessory enzyme whose activity is required for optimal production of Pfa synthase derived end products including omega-3 PUFAs and long-chain olefin hydrocarbons.

## Author Contributions

MA, CS, and EA conceived the idea, designed the project, and contributed to the discussion and critical writing. MA and CS carried out the experiments and collected and analyzed the data. MA wrote the manuscript.

## Conflict of Interest Statement

The authors declare that the research was conducted in the absence of any commercial or financial relationships that could be construed as a potential conflict of interest.
