# Supplementary material for: Linkage of Marine Bacterial Polyunsaturated Fatty Acid and Long-Chain Hydrocarbon Biosynthesis
Source: Front Microbiol. 2019 Apr 3;10:702. doi: 10.3389/fmicb.2019.00702 (PMC6463001; doi:10.3389/fmicb.2019.00702)
Supplement: Supplementary file 1 [file Data_Sheet_1.PDF]

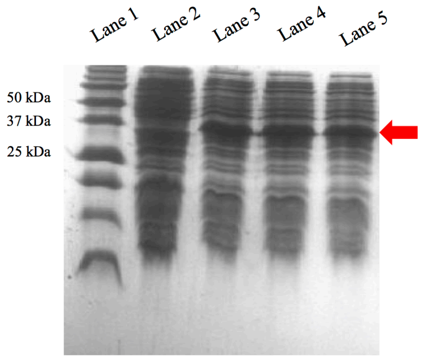

Supplementary Figure 1 SDS-PAGE of whole cell lysates of MAE21 containing pMA63 grown at 15°C; Lane 1, protein standard ladder; Lane 2-5; 0, 0.001, 0.05, and 0.1% L-Arabinose. Arrow indicates OleA band at approximately 38kDa.

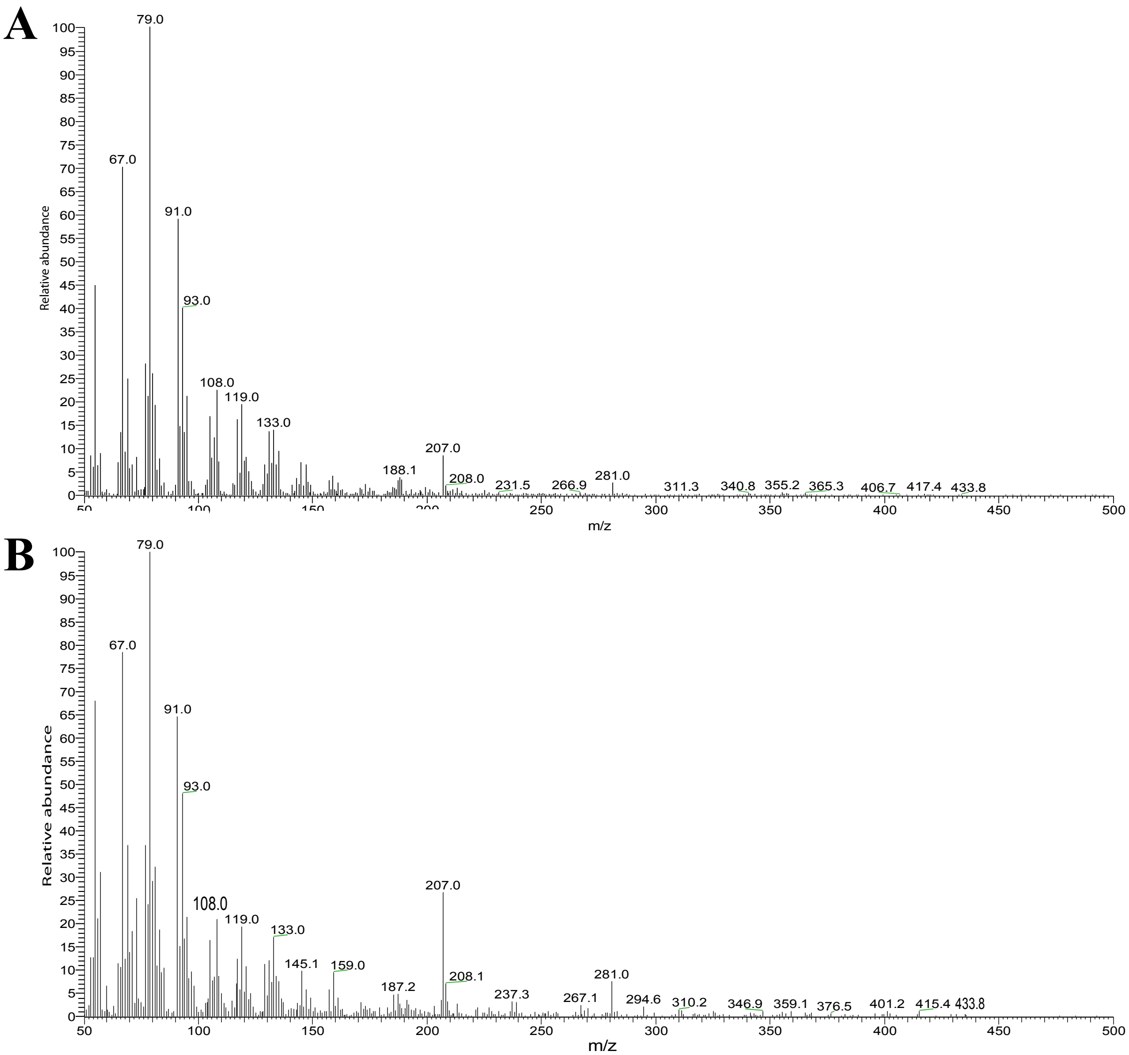

Supplementary Figure 2 Mass spectra of 31:8 ketone product produced by OleA. (A) Spectra of 31:8 ketone being produced by *Shewanella oneidensis* MR-1 *ΔoleABCD* with pOleA. (B) Spectra associated with peak at 23.1min from FIGURE 3B in main text.

Supplementary Table 1 - Primers used in this study, restriction sites underlined

Primer name	Sequence 5'→3'
MR1 SO1256 5'O	TGACTCATATGGCCCACGTGATATGGGTTAC
MR1 SO1256 5'I	GACTGGCTTAGGTCGTCTGGCGGCTAAATTGCGGCATAAAA
MR1 SO1256 3'I	GCCAGACGACCTAAGCCAGTCCCCATGCCTGCAGAGTTAAT
MR1 SO1256 3'O	TGACTGAGCTCGCGGGAATTTCTGTTTGTGT
MR1 orf6 del ver F	GCTCAGAAGGCTTGATTTGG
MR1 orf6 del ver R	CTGCAACGTCACAGAACCAT
SS9 orf6 5'O	TGACTGGTACCCCCGAAAGTACGCCTAAACA

SS9 orf6 5'I	GACTGGCTTAGGTCGTCTGGCTTGCACTGGGTGGTGATAAA
SS9 orf6 3'I	GCCAGACGACCTAAGCCAGTCTGGCTTTTAGAGCGTTTTCC
SS9 orf6 3'O	TGACTGAGCTCAGGAGGCACTTACCATCCCT
SS9 orf6 del ver F	ATCCATAATGCCCATGGAAA
SS9 orf6 del ver R	TCGCAATTGGCATGTCTTTA
pKT231 orf6 F	GGGGCTAAACGGAATTCTAACT
pKT231 orf6 R	TACGATGGATCCAGGGCTATCAATTGGTTGGA
pBAD24 Spea oleA F	CGATCGCTAGCAGGAGGGCAGTTTCATGAAATATTCCCG
pBAD24 Spea oleA R	ACGATCCTGCAGGTAGGCTGGTTTCAATTACC
pBAD24 SS9 orf6 F	TACGATGAATTCAGAGTTGTTGCAGCAATGAG
pBAD24 SS9 orf6 R	TATAGTCTAGATTAATCAGCCATCATCGAAG
pBAD24 Spea orf6 F	ACGATCGAATTCACTATGTCGCAAACTCATAC
pBAD24 Spea orf6 R	ACGATCCTGCAGAAGGTAAGTCGGTTATGTCG
OleA C123A mut F	GATATCAGCAACGCTGCTCTTGGTGTGCTTTCA
OleA C123A mut R	TGAAAGCACACCAAGAGCAGCGTTGCTGATATC
Spea pfaE F	ATGGAGGCTGTTGAGTTTGG
Spea pfaE R	GGCTAAGCCCAATCCCTTAG
Spea pfaD F	CGAGATGGGCGTTAAGCTAC
Spea pfaD R	GCGCTCATTAAAGTGTGCAA

Supplementary Table 2- *S. oneidensis* MR-1 fatty acid profiles at 15°C

	MR1[a]	MAS1[a]	Δ*oleABCD*[a]
12:0	2.17 ± 0.28	2.26 ± 0.13	2.28 ± 0.05
13:0	3.66 ± 1.05	3.12 ± 0.34	3.07 ± 0.42
13:0iso	0.59 ± 0.13	0.56 ± 0.09	0.53 ± 0.03
12:0 3-OH	1.61 ± 0.24	1.72 ± 0.05	1.76 ± 0.48
13:0an	1.01 ± 0.26	0.77 ± 0.08	0.79 ± 0.13
14:1	0.36 ± 0.02	0.37 ± 0.02	0.35 ± 0.00
14:0	1.92 ± 0.37	1.91 ± 0.19	1.52 ± 0.03
14:0 3-OH	2.12 ± 0.30	1.82 ± 0.11	1.91 ± 0.53
15:0iso	30.29 ± 2.28	28.20 ± 3.11	27.15 ± 2.43
15:0an	2.80 ± 0.14	3.05 ± 0.78	2.61 ± 0.42
16:1	25.40 ± 0.85	30.43 ± 4.88	29.35 ± 0.65
16:0	8.78 ± 2.73	7.89 ± 2.11	8.51 ± 1.32
17:0	1.43 ± 0.27	1.18 ± 0.08	1.31 ± 0.02
17:0cyc	8.73 ± 1.29	8.20 ± 1.21	9.14 ± 0.64
17:0iso	1.11 ± 0.38	1.15 ± 0.58	1.11 ± 0.45
18:1	7.46 ± 1.17	7.02 ± 0.27	7.69 ± 1.07
18:0	0.30 ± 0.12	0.29 ± 0.02	0.35 ± 0.08
20:5	0.26 ± 0.04	0.06 ± 0.01	0.55 ± 0.04
UFA/SFA	0.50	0.61	0.61

[a] Values are the mean ± standard deviation from at least three independent replicate cultures

Supplementary Table 3-Fatty acid profiles of *E.coli* strain MAE21 grown at 15°C

	JW1794 pCC2FOS[a]	MAE21[a]	MAE21 pMA47[a]	MAE21 pMA48[a]
12:0	1.68 ± 0.74	1.41 ± 0.13	1.46 ± 0.24	1.35 ± 0.31
14:0	4.67 ± 1.13	4.57 ± 0.06	4.59 ± 0.13	4.64 ± 0.23
14:0 3-OH	1.67 ± 0.42	3.54 ± 0.41	3.49 ± 0.41	3.69 ± 0.46
16:0	29.00 ± 4.86	30.74 ± 0.76	30.31 ± 0.95	29.40 ± 1.06
16:1	19.03 ± 2.32	9.02 ± 1.83	10.77 ± 1.08	9.69 ± 1.11
18:1	43.11 ± 4.10	27.72 ± 0.89	28.51 ± 2.53	27.90 ± 1.07
18:0	0.85 ± 0.23	0.34 ± 0.58	0.33 ± 0.57	0.33 ± 0.57
20:5	0.00 ± 0.00	21.46 ± 2.44	19.93 ± 3.13	22.25 ± 1.03
UFA/SFA	1.64	1.43	1.47	1.52

[a] Values are the mean ± standard deviation from at least three independent replicate cultures

Supplementary Table 4- Fatty acid profiles of SS9R and MAP1 as a function of temperature and pressure

	SS9R[a]			MAP1[a]		
	15°C	4°C	30MPa, 15°C	15°C	4°C	30MPa, 15°C
12:0	4.06 ± 1.47	4.20 ± 1.42	1.96 ± 0.16	3.93 ± 1.29	3.87 ± 1.21	1.79 ± 0.29
14:0	4.17 ± 1.09	3.25 ± 0.62	2.84 ± 0.28	4.02 ± 1.38	2.46 ± 0.46	2.65 ± 0.35
14:1	3.24 ± 1.07	2.69 ± 0.68	0.74 ± 0.04	2.58 ± 0.35	3.05 ± 0.92	0.64 ± 0.02
16:0	23.04 ± 3.34	22.56 ± 2.58	26.45 ± 0.87	21.75 ± 4.13	19.45 ± 0.80	26.48 ± 1.33
16:1	43.29 ± 2.51	41.91 ± 0.29	49.42 ± 1.17	48.48 ± 0.73	50.06 ± 2.37	52.06 ± 2.25
12:0 3-OH	1.79 ± 1.14	2.32 ± 0.87	1.22 ± 0.13	1.52 ± 0.45	1.84 ± 1.15	1.59 ± 0.30
18:0	0.63 ± 0.20	0.07 ± 0.12	10.26 ± 0.39	0.68 ± 0.03	0.07 ± 0.13	12.77 ± 0.52
18:1	11.47 ± 3.23	9.39 ± 0.45	0.44 ± 0.39	13.05 ± 0.66	13.19 ± 1.75	0.38 ± 0.66
20:5	4.98 ± 1.18	7.83 ± 0.52	6.67 ± 0.22	1.27 ± 0.29	2.33 ± 0.26	1.64 ± 0.28
UFA/SFA	1.869	1.908	2.039	2.049	2.478	2.040

[a] Values are the mean ± standard deviation from at least three independent replicate cultures at the indicated culture condition

Supplementary Table 5- Fatty acid profiles of *pfaT* complementation strains at 15°C

	SS9R pKT231[a]	MAP1 pKT231[a]	MAP1 pMA20[a]
12:0	2.73 ± 0.25	2.27 ± 1.13	2.16 ± 0.47
14:0	5.19 ± 0.40	4.36 ± 0.01	3.44 ± 1.88
14:1	3.28 ± 0.21	2.48 ± 0.02	2.10 ± 0.94
16:0	20.20 ± 1.50	21.08 ± 0.22	21.94 ± 1.30
16:1	46.40 ± 3.72	49.44 ± 0.32	51.27 ± 3.46
12:0 3-OH	1.55 ± 0.16	1.61 ± 0.18	0.98 ± 0.49

18:0	1.87 ± 1.41	1.20 ± 0.04	0.82 ± 0.33
18:1	13.23 ± 0.85	15.67 ± 0.39	12.95 ± 0.39
20:5	1.61 ± 0.17	0.62 ± 0.08	2.73 ± 0.29
UFA/SFA	2.05	2.38	2.35

[a] Values are the mean ± standard deviation from at least three independent replicate cultures
